# Chanalyzer: A Computational Geometry Approach for the Analysis of Protein Channel Shape and Dynamics

**DOI:** 10.3389/fmolb.2022.933924

**Published:** 2022-07-25

**Authors:** Andrea Raffo, Luca Gagliardi, Ulderico Fugacci, Luca Sagresti, Simone Grandinetti, Giuseppe Brancato, Silvia Biasotti, Walter Rocchia

**Affiliations:** ^1^ Istituto di Matematica Applicata e Tecnologie Informatiche “E. Magenes”, Consiglio Nazionale delle Ricerche, Genova, Italy; ^2^ CONCEPT Lab, Istituto Italiano di Tecnologia, Genova, Italy; ^3^ Scuola Normale Superiore, Pisa, Italy; ^4^ Istituto Nazionale di Fisica Nucleare (INFN), Pisa, Italy; ^5^ Consorzio Interuniversitario per lo sviluppo dei Sistemi a Grande Interfase (CSGI), Sesto Fiorentino, Italy; ^6^ Dipartimento di Ingegneria Civile ed Industriale, Università di Pisa, Pisa, Italy

**Keywords:** channel and pore characterization, computational geometry, molecular surface, molecular dynamics, skeletonization, alpha shapes theory, ion channels

## Abstract

Morphological analysis of protein channels is a key step for a thorough understanding of their biological function and mechanism. In this respect, molecular dynamics (MD) is a very powerful tool, enabling the description of relevant biological events at the atomic level, which might elude experimental observations, and pointing to the molecular determinants thereof. In this work, we present a computational geometry-based approach for the characterization of the shape and dynamics of biological ion channels or pores to be used in combination with MD trajectories. This technique relies on the earliest works of Edelsbrunner and on the NanoShaper software, which makes use of the alpha shape theory to build the solvent-excluded surface of a molecular system in an aqueous solution. In this framework, a channel can be simply defined as a cavity with two entrances on the opposite sides of a molecule. Morphological characterization, which includes identification of the main axis, the corresponding local radius, and the detailed description of the global shape of the cavity, is integrated with a physico-chemical description of the surface facing the pore lumen. Remarkably, the possible existence or temporary appearance of fenestrations from the channel interior towards the outer lipid matrix is also accounted for. As a test case, we applied the present approach to the analysis of an engineered protein channel, the mechanosensitive channel of large conductance.

## 1 Introduction

Ion channels (Kew and Davies, 2009; Lemoine et al., 2012) are a biological class of prominent pharmacological importance and are targets for over 20% of drugs on the market. Moreover, to expand their use beyond the natural one, ion channels have been successfully modified to confer them new artificial gating mechanisms through the combination of molecular biology and protein engineering (Banghart et al., 2006). In addition to membrane proteins, channel-like passages can be also found in other relevant intracellular proteins, as in ferritin where channels regulate ion uptake and release (Chandramouli et al., 2016). Therefore, characterizing the structural and dynamic features of ion channels can significantly improve our understanding of their functioning and unveil the more subtle details of their mechanism, which are often elusive to experimental observations. In this context, molecular dynamics (MD) simulations have proven very valuable to investigate channel behavior with atomistic detail, thus integrating biological and crystallographic information and helping achieve a more comprehensive understanding of channel function (Treptow and Klein, 2012). As an example, MD simulations have been fruitfully applied to describe pathways and barriers for ion translocation in a pentameric ligand-gated ion channel (Di Maio et al., 2015) or to gain new insights into the peculiar gating mechanism of an engineered protein (Chandramouli et al., 2015), the mechanosensitive channel of large conductance (MscL) equipped with a light-triggered gating, as originally proposed by Feringa and coworkers (Koçer et al., 2005). MD also proved to be instrumental in the characterization and design of biological pore constructs for biotechnological applications (Spitaleri et al., 2021).

Here, we propose a novel computational tool for the automatic recognition and structural characterization of a protein channel from a given MD trajectory, rooted in the alpha shape complex analysis and the notion of discrete flow as described in (Edelsbrunner et al., 1998), *see* more details in Section 2.1. Alpha shape theory is also at the basis of how the NanoShaper software builds the protein Solvent-Excluded Surface (SES) and finds cavities and pockets (Decherchi and Rocchia, 2013). This method enriches the capabilities of NanoShaper to identify and characterize cavities in molecular structures (Decherchi et al., 2018) and is focused more specifically on the identification of permanent or transient channels formed within a protein, from which it was dubbed Chanalyzer. Notably, it does not require predefined user parameters for channel identification, such as the notion of membrane plane, it is numerically robust and well-suited for analyzing a large collection of molecular configurations as issued from extended MD simulations of biological systems. In addition, the method supports a detailed geometric analysis based on the concepts of skeleton and centerline and identifies both channels ends through the use of graph-based techniques. The identification of the channels is computationally efficient and a proximity strategy has been adopted to accelerate the calculation of channel entrance and exit from one MD frame to the following one. Interestingly, its extensive geometric characterization allows for the identification of the channel main axis, but also of possible ancillary tunnels and potential fenestrations facing the lipid membrane. It also provides a geometric approximation of the local section orthogonal to the central axis of the lumen, to more easily identify symmetry-breaking configurations and anomalies. To validate the present approach and to show the complete set of geometrical information that it provides, we analyzed a number of MD trajectories of an engineered protein channel, the mechanosensitive channel of large conductance (MscL). Overall, results show a very good agreement with previous calculations of channel local radius, as well as the provisioning of new information thus supporting the routine use of Chanalyzer for the detailed study of protein channels in a large variety of biological systems.

## 2 Methods

### 2.1 Preliminary Concepts on Alpha Shape Theory

A popular representation of macromolecules is the union of possibly overlapping spherical balls, each of them representing an atom. Using computational geometry concepts, the relative distances among the atoms are captured by the Voronoi diagrams (Voronoi, 1908) and Delaunay simplices (Delaunay et al., 1934). These concepts are fundamental for defining and delimiting both the space occupied by the molecule and its complement (Edelsbrunner and Mücke, 1994). The illustration of the Delaunay triangulation (Dt) of a simplified molecule, represented in 2D as a set of disks of the same radius, is shown in Supplementary Figure S1 in the supplemental material (disks are in light gray). The Dt triangulates the convex hull of the centers of the disks, representing the atoms. It reports a *complex*, which is basically a set of triangles or tetrahedra in the space. The part of the complex that corresponds to the molecule is the so-called *alpha shape* and corresponds to triangles that cover only regions inside the union of the disks and, in degenerate cases, contains also segments and isolated points. In the figure, the alpha shape is represented by gray triangles and bold black edges. The complement can be organized into connected components. With respect to the figure, there are three components, the largest one is represented in light blue. The small black arrows indicate the edges of the complement external to the alpha shape. These edges can be envisaged as part of a degenerate triangle having the third vertex at the infinity. Starting from these edges, it is possible to define a direction of visit for the component, the so-called discrete flow, and to determine how deep that component is. Components without external edges – such as the one in light red on the right – correspond to internal voids; components with one external edge (mouth) correspond to pockets; components with two mouths correspond to channels.

### 2.2 Geometric Processing

#### 2.2.1 Trajectory Post-Processing

The trajectory is converted from the original *dcd* to the multiple *pdb* format using the VMD tool (Humphrey et al., 1996), after excluding water molecules and ions. Then it is split into individual frames and finally annotated with the atomic radii information. Finally, only the information on atom centers and radii is retained in a.xyzr file, which is the standard input of NanoShaper (Decherchi and Rocchia, 2013).

#### 2.2.2 Channel Identification

**FIGURE 1:**
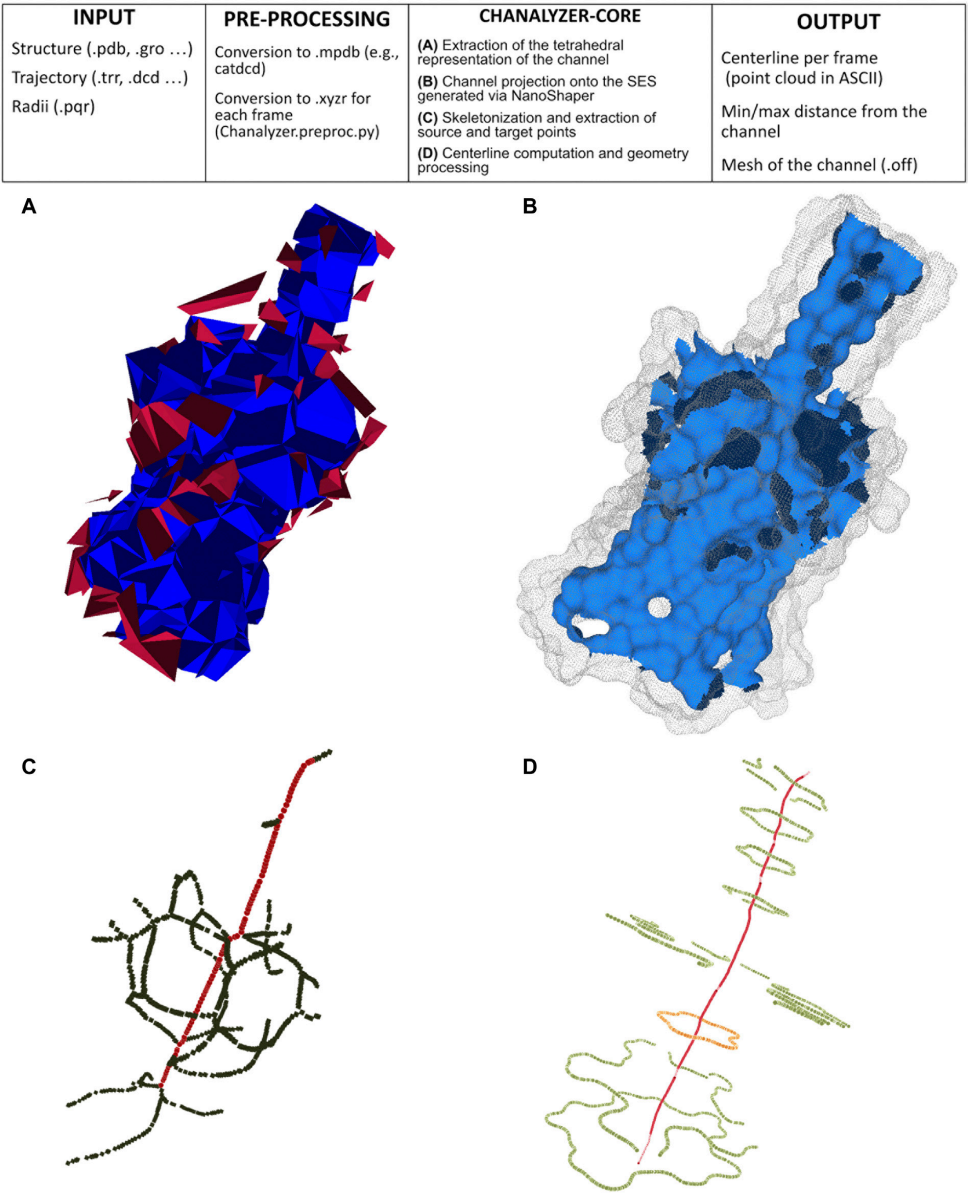
Pipeline of Chanalyzer. At the top, the basic flow is described. **(A)** Identification of the connected components of tetrahedra that are not entirely inside the SES. Each component represents a cavity (in blue the largest one, ascribed to the channel, in red the other ones, which are discarded); **(B)** the intersection of the blue component with the SES identifies the channel surface; **(C)** skeletonization (in green) revealing a footprint of the pentameric structure and the source-target path (in red); **(D)** centerline in red and several sections (in orange the central one).

We build our method to identify channels on the alpha shape theory (Edelsbrunner and Mücke, 1994). A molecule is here modeled as a collection of three-dimensional balls, one ball per atom; since the radius varies with the different atoms, we build their weighted Delaunay triangulation (wDT) using the routines in (The CGAL Project, 2013). We then obtain a *simplicial complex* whose nodes correspond to the atoms and volumetric elements are tetrahedra. As a convention, it is assumed that the faces of the convex hull of the wDT are virtually connected to a point at infinity. We characterize this structure using the discrete-flow procedure as detailed in (Edelsbrunner et al., 1998). The discrete-flow aims at identifying the tetrahedra of the simplicial complex that are in the complement of the molecule and is defined on the basis of geometric considerations on how much a tetrahedron is “obtuse” and its circumcenter spans over external tetrahedra. In more detail, starting from the tetrahedra that include the infinity point (those tetrahedra are external by definition), the flow induces a partial ordering between couples of adjacent tetrahedra. At this point, using a graph depth-first visiting scheme driven by the discrete-flow ordering, we identify the tetrahedra that descend from the infinity: each arc of the graph identifies a connected component of tetrahedra that is a potential cavity of interest, as discussed in (Liang et al., 1998). Figure 1A visualizes the connected components of tetrahedra obtained during the graph visiting.

The connected component having the largest volume, shown in blue in Figure 1A, corresponds to the volumetric approximation of the channel. To estimate geometric properties, this tetrahedral approximation is projected onto the molecular surface, here built *via* NanoShaper as a triangular mesh approximation of the SES. The SES triangular mesh is processed to keep only those triangles having the barycenter inside the tetrahedral approximation of the channel. This extraction is based on the query of a k-d tree. It is worth noting that other molecular surface representations and other software to compute the MS could in principle be employed too; we here rely on NanoShaper as it also bases the MS computation on the SES definition *via* alpha shape theory and weighted Delaunay triangulations.

#### 2.2.3 Geometric Characterization

The outcome of Section 2.2.2 is a portion of the SES that roughly corresponds to the channel, *see* an example in Figure 1B. In a generic case, such a surface is expected to be roughly tubular in shape but, in some particular cases, it can present bi- or multi-furcations, whose biological or biophysical implications might need some attention. To characterize such a surface portion, we firstly extract the mean curvature flow skeleton (Tagliasacchi et al., 2012) using its CGAL implementation, which is an approximation of the medial axis of the channel. Even if intuitive and very popular in computer graphics, the skeleton is strongly sensitive to surface perturbations and corners, as visible in Figure 1C. Therefore its robust identification requires particular caution.

##### Skeleton Identification and Pruning

The skeleton of the cavity previously extracted *via* CGAL does not present a linear structure but it may contain several ramifications and ancillary paths preventing a clear identification of the channel and its main entrances (*see* Figure 1C). In order to overcome this limitation, we adopted a pruning procedure extracting from the skeleton the path that best represents the main direction along which the channel structure develops. Once this is done, we can also locate the two main entrances, discriminating them from possible lateral alternatives. This pruned representation of the skeleton is extracted by retrieving in the skeleton graph a path with the property of maximizing both length and straightness. In order to achieve this, a score *s*(*γ*) is defined for each path *γ* of the graph as follows:

$$s\left(\gamma \right)≔\frac{length{\left(\gamma \right)}^{2}}{tortuousness\left(\gamma \right)},$$ (1)

where *length*(*γ*) is the number of edges that compose *γ* and *tortuousness*(*γ*) is a positive real number close to zero when *γ* is rectilinear and steadily increases as the path loses its rectilinear behavior. It is calculated as the average distance of the nodes of *p* from the straight line connecting its start and end nodes. Thus, paths assuming high score values are both straight and long while curved/short paths present low score values. The pruning algorithm considers the shortest paths connecting all the pairs of vertices of the skeleton graph and computes the score value for each of them. Then, the shortest path *γ* having the maximum score value is elected as the core skeleton of the channel and adopted as input to the next steps of the channel identification pipeline. In Figure 1C we superimpose the output of the pruning algorithm, red dots, to the curve skeleton obtained with CGAL.

##### Centerline Computation

To study tubular geometries with complex morphologies we here employ the Vascular Modeling ToolKit (VMTK) (Piccinelli et al., 2009), a standard software package for the geometric analysis of vessels segments. Given source and target points, VMTK can provide a number of useful quantities about the global morphological properties, such as the unit vectors of local Frenet-frames of the centerline which, in turn, allow to compute curvature and torsion. The skeleton pruning step allows Chanalyzer to focus on the channel axis and, potentially, to consider secondary entrances.

##### Extraction of Visible Contour of a Channel

**FIGURE 2:**
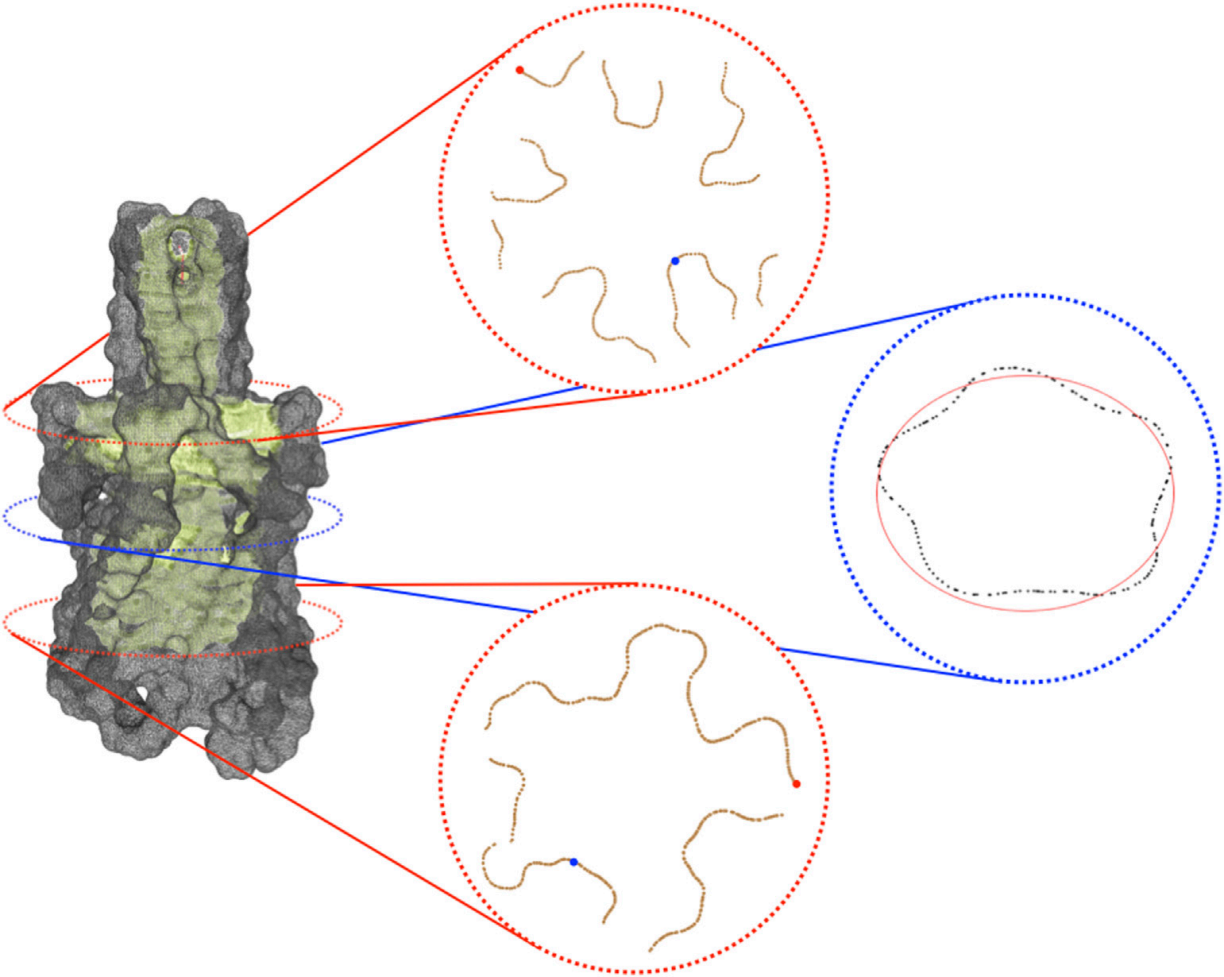
Visible contour of a channel. On the left, is the visible contour of the channel (in light green) and the molecular surface (in grey). On the right, three sections of the visible contour: on the top, the section coinciding with the bifurcation of the skeleton; in the middle, the central section of the channel; at the bottom, a section clearly revealing the pentalobated nature of the channel. In each section, we spotlight some of the geometric features provided by the proposed approach. Specifically, in the top and in the bottom sections, the closest and the farthest points to the centerline are represented in blue and red, respectively. In the middle section, the ellipse (depicted in red) that best fits it is shown. Its knowledge allows retrieving further information about the local channel shape, such as its eccentricity.

The previous steps of the procedure allow projecting the identified channel on the molecular surface. In order to better analyze the channel and compute information about its shape, it can be useful to consider, rather than all the portion of the surface identified as composing the channel, just the part of it which is visible from the centerline. The extraction of this visible contour of the channel is achieved by clipping the channel surface with planes that are orthogonal to the centerline itself. More specifically, given a point *p* of the centerline, *π*_*p*_ is defined as the plane passing through *p* and orthogonal to *t*_*p*_ where *t*_*p*_ is the tangent vector of the centerline at the point *p*. Intersecting *π*_*p*_ with the channel surface, the section *S*_*p*_ of the channel at level *p* is retrieved. One can expect that such a section is a simple and closed curve but, in practice, *S*_*p*_ can also consist of multiple connected components not all visible from the point *p*. In order to extract from *S*_*p*_ the portion visible from *p*, we consider a suitable sampling of the rays starting from *p* and belonging to *π*_*p*_ and for each of them, we return the points of *S*_*p*_ intersecting the selected ray and visible from *p*. More properly, we represent the points of *S*_*p*_ as a graph *G* whose vertices are the points of *S*_*p*_ and whose edges consist of the pairs of points of *S*_*p*_ closer than a given threshold. Given a ray *r*, we select, among the points of *S*_*p*_ close to *r*, the one which is the closest to *p* and then we return the connected components of *G* containing such a vertex. The collection of connected components retrieved by varying the ray will represent a suitable pruning of section *S*_*p*_ and a good candidate for representing the contour of the channel at the level of *p*. The knowledge of the sections of the channel contour opens various possibilities for visualizing and analyzing the shape of the channel itself. Finally, in Figure 1E we depict the centerline obtained with VMTK along with some channel sections. For instance, for each point *p* of the centerline, it is possible to evaluate the closest and the farthest points of the contour section (and the corresponding distances) to *p* as well as evaluate the symmetry, the radius, and the shape of the various contour sections (*see* Figure 2), for instance fitting a contour section with an elliptical template using the Hough transform (Romanengo et al., 2019).

#### 2.2.4 From Individual Structures to Trajectory Analysis

In Sections 2.2.2 and 2.2.3 we described how to analyze an individual structure. This procedure can be fruitfully extended to many frames extracted from an MD trajectory, taking advantage of the fact that the displacement of the molecule between two consecutive frames is limited. Therefore, we perform the full procedure of skeleton identification only on the first frame. Then, for the (*i* + 1)-th frame, we use an initial guess regions for the entrances the borders of the surface closest to the channel’s entrances frame *i*. If we notice a significant discrepancy between the centerlines of two consecutive frames, we reinitialize the procedure.

#### 2.2.5 Computational Complexity

The theoretical computational costs for extracting the simplicial complex and identifying the connected components between which to identify the channel are both *O*(*n* log*n*) on the number of atoms considered. For the geometric characterization of the channel, the cost is: *O*(*t* log*t* + *m* ⋅ *s*) for the identification of the channel surface where *t* is the number of vertices of the SES, *m* is the number of atoms of the channel, *s* is the number of vertices of the channel mesh; the skeleton costs *O*(*s* ^2^), while the centerline and its characterization occur in *O*(*s* log*s*) + *O*(*s* ⋅ *r*) operations where *r* is the number of points where we estimate the centerline.

The worst-case complexity of the skeleton pruning is *O*(*v* ⋅ *e* + *v* ^2^) where *v* and *e* are the number of vertices and edges of the skeleton, respectively.

The extraction of the visible contour of a channel for a point *p* of the centerline has worst-case complexity *O*(*s* + *d* ⋅|*S*_*p*_|) where *d* is the number of directions or rays on which the visibility is evaluated and |*S*_*p*_| is the number of points which the section of the channel surface orthogonal to the centerline at *p* consists of. For all the points of the centerline, the complexity of such a procedure is *O*(*s* ⋅ *r* + *d* ⋅|*S*|⋅ *r*) where *r* is the number of points of the centerline and |*S*| is the maximum number of points composing a section. Assuming that the number of SES vertices of the protein goes as *O*(*n* ^2^) and that the size of the channel is somehow limited, a rough estimate of the overall complexity is *O*(*n* ^2^ *log*(*n*)).

### 2.3 Molecular Dynamics Simulations

The studied molecular systems are four variants of the engineered MscL channel, a pentameric protein channel, originally investigated in (Chandramouli et al., 2015) (PDB code 2OAR). Each variant differs from the others in the way residue 21 is functionalized among the five subunits. In this functionalization, a photo-activating ligand, namely the 6-nitroveratryl alcohol, which splits into 6-nitrosoveratyl aldehyde and a free acid upon light irradiation, was attached through a Cysteine-selective alkylating reagent to the residue 21 of each protein monomer (more details can be found in (Chandramouli et al., 2015)). Concerning the MD simulations, the adopted force field is CHARMM (v.27 (MacKerell et al., 1998)) for the protein and (v.36 (Klauda et al., 2010)) for the lipid. The parameters for the photo-activating ligand have been computed through QM calculations at the DFT level of theory. The water model is TIP3P, while the ionic concentration of *K* ^+^ and *Cl* ^−^ is set to 1 M. Equilibration is carried out for about 10 ns in the NpT ensemble, followed by the production run according to the NVT ensemble under normal conditions (T = 300 K). For nonbonded interactions, a cutoff of $12\overset{{}^{\circ}}{A}$ is used. All simulations are performed with periodic boundary conditions, treating the long-range electrostatic interactions with the Particle Mesh Ewald (PME) algorithm (Darden et al., 1993).

## 3 Results

**FIGURE 3:**
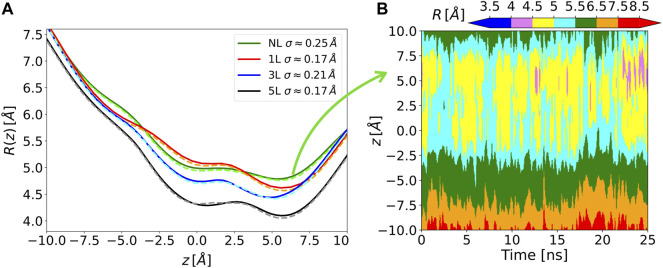
**(A)** Solid lines, time-averaged channel radius along with the axial z position for each of the considered systems as obtained by Chanalyzer with associated SD (in the legend). Dashed lines, the same radius derived *via* the HOLE software. **(B)** Example of the dynamical behavior for the no-ligand system. The colormap is associated with the instantaneous value of the radius, as returned by Chanalyzer.

The Chanalyzer approach is applied to the analysis of the MscL system in order to compare the present analysis to the one originally carried out (Chandramouli et al., 2015). In that work, a variable number of modifications of the MscL channel was applied to generate corresponding molecular models by attaching a photo-activating ligand at residue 21 to the five monomers of the protein, as experimentally tested by Feringa and collaborators (Koçer et al., 2005). We focused on four of these functionalized systems, namely the NL, 1, 3, and 5L, having 0, 1, 3, and 5 photo-activating ligands, respectively. In the analysis reported in (Chandramouli et al., 2015), snapshots of the MD trajectory were superimposed and fed to the HOLE software (Smart et al., 1996) after removing the side chains, to measure the radius of the channel of the different generated models. This was aimed at highlighting the symmetry breakage and at confirming the progressive engineered expansion of the channel radius with the sequential addition of negative charges upon photo-ligand removal. This effect is apparent in Figure 3 where the computed average radii evaluated along the longitudinal channel axis (i.e., at different *z* values) by the present approach and by the HOLE software (Smart et al., 1996) are shown. Averages are performed over about 150 MD configurations for each considered system and a re-binning procedure is used along the *z*-axis. The agreement between Chanalyzer (solid lines) and HOLE (dashed lines) data is extremely good, with results basically indistinguishable in most regions and abundantly within the standard deviation in correspondence of local minima and saddle points. The observed systematic shift in pore radius (from 5L to NL) supports an increasing role of the residual charges, thus confirming the previously observed charge-mediated MscL gating (Birkner et al., 2012).

**FIGURE 4:**
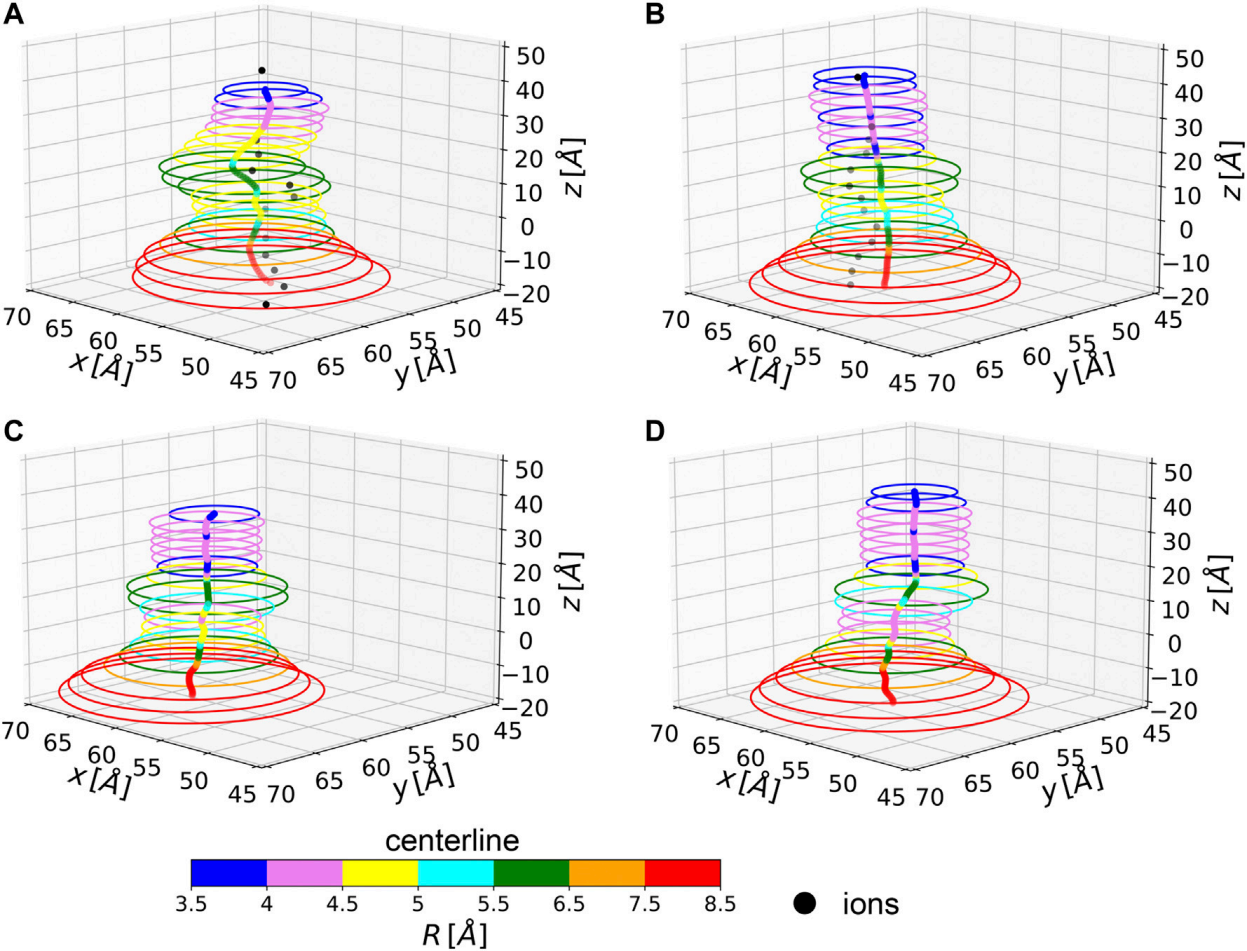
Average centerlines. Colors code for the size of the associated radius. Black dots are average ion positions for the permeating configurations. From top to bottom and left to right: **(A)** NL, **(B)** 1L, **(C)** 3L, and **(D)** 5L.

In two of the simulated systems, namely NL and 1L, potassium ion percolation was also observed. It is therefore interesting to compare how close are ion permeation paths to the centerlines of the corresponding MscL channels. Note, however, that ion translocation pathways can be affected by local and specific steric or electrostatic effects induced by residue side chains, not accounted for in our evaluation of the centerline which is based only on backbone atoms. In Figure 4, the centerlines, as returned by Chanalyzer, and the average trajectories followed by the *K* ^+^ ions in the NL and 1L systems are depicted. In the latter case, the average is carried over the multiple MD configurations and a re-binning is performed along the *z*-axis. The general trend is that in those locations where the radius is smaller, as expected, ions are more constrained towards the centerline. However, around $z=10\overset{{}^{\circ}}{A}$ the ions tend to be more displaced towards the channel walls as compared to other locations with similar radii. This is likely a consequence of the net electrostatic attraction of the charged residues, upon photo-ligand removal. Interestingly, this suggests that a systematic comparison between centerlines and ion trajectories could be used as an indirect way to probe the local interactions between ions and residues along the channel and can be helpful in suggesting preferential mechanisms affecting translocation.

## 4 Discussion

We presented here the application of the Chanalyzer geometric approach to the analysis of the channel morphology and dynamics of four differently functionalized forms of the MscL system, as a test case. Computational geometry has been already exploited to study the details of molecular structures. For instance, cavities and tunnels arising at the molecular surface of a protein have been studied with the NanoShaper software (Decherchi et al., 2013; Decherchi and Rocchia, 2013). In the field of protein channels, a popular analysis tool is provided by the HOLE software (Smart et al., 1996), which finds the maximum radius of a sphere centered within the channel starting from a given point (provided by the user to be inside the channel), so as that it does not overlap with the van der Waals interior surface of the pore and makes that sphere proceed and adapt its size throughout the channel, assumed to be nearly rectilinear. Successively, two tools have been developed by the same group, namely CAVER (Petřek et al., 2006) and its improved version, MOLE (Petřek et al., 2007), to explore routes between protein clefts and cavities. CAVER’s underlying algorithm is based on a skeleton search using a three-dimensional grid. Finally, MolAxis (Yaffe et al., 2008), a more recent tool also based on alpha shape theory, was successfully applied to the 5HT3 receptor (Di Maio et al., 2015) to identify lateral ion channels besides the central longitudinal one. However, MolAxis still strongly relies on user parametrization and can suffer from method-specific artifacts and approximations. While further improvements to the Chanalyzer project are still needed and its development is currently ongoing, it already sets up a framework that enables the accurate evaluation of several channel features that start from the purely geometric analysis but can easily integrate other relevant physico-chemical information, e.g. the chemical nature (i.e., atom identification) of the pore lumen as it inherits the properties of the SES calculated by NanoShaper. Remarkably, the fact that Chanalyzer does not need user-specific parameterization, such as a predetermined direction of the channel axis, does represent a clear advantage in the treatment of those cases where the geometrical shape is not predominantly tubular and may present bi- or multi-furcations, as well as ancillary pathways towards the surrounding lipid matrix. These so-called fenestrations may have relevant biological or biophysical implications still not well known (Jorgensen et al., 2016). For such reasons, we believe that biophysical modeling can significantly benefit from user-friendly and versatile geometric approaches, such as Chanalyzer.

## Data Availability

The datasets presented in this study can be found in online repositories. The names of the repositories and accession numbers can be found on github at: https://github.com/rea1991/Chanalyzer and: https://github.com/concept-lab/mpdb2xyzr.git or on Zenodo with DOIs:1 0.5281/zenodo.6610045 and 10.5281/zenodo.6509652.
