# Supplementary material for: Chanalyzer: A Computational Geometry Approach for the Analysis of Protein Channel Shape and Dynamics
Source: Front Mol Biosci. 2022 Jul 25;9:933924. doi: 10.3389/fmolb.2022.933924 (PMC9358003; doi:10.3389/fmolb.2022.933924)
Supplement: Supplementary file 1 [file DataSheet1.PDF]

Supplementary Material

1 SUPPLEMENTARY FIGURES

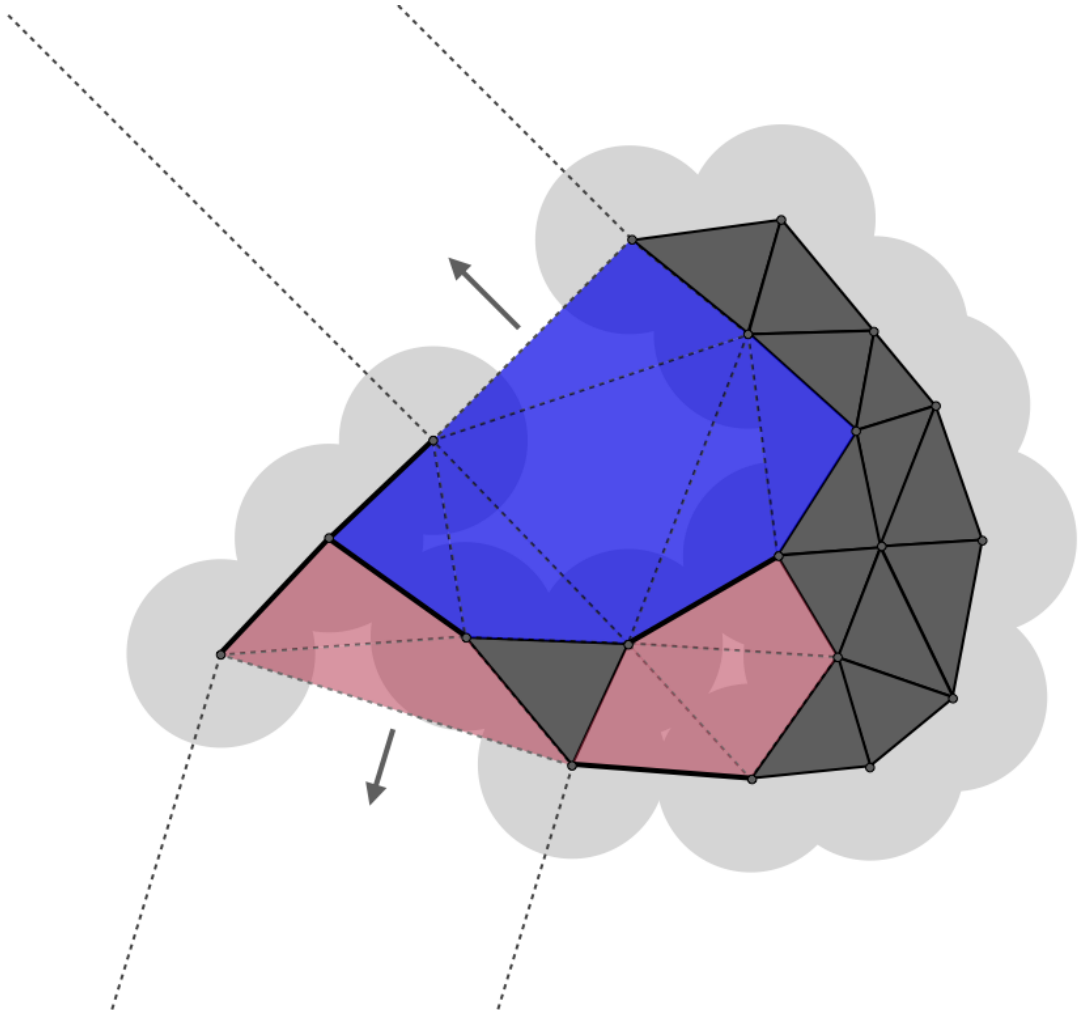

Figure S1. Schematic representation of how cavities are identified via the Alpha Shapes theory

.

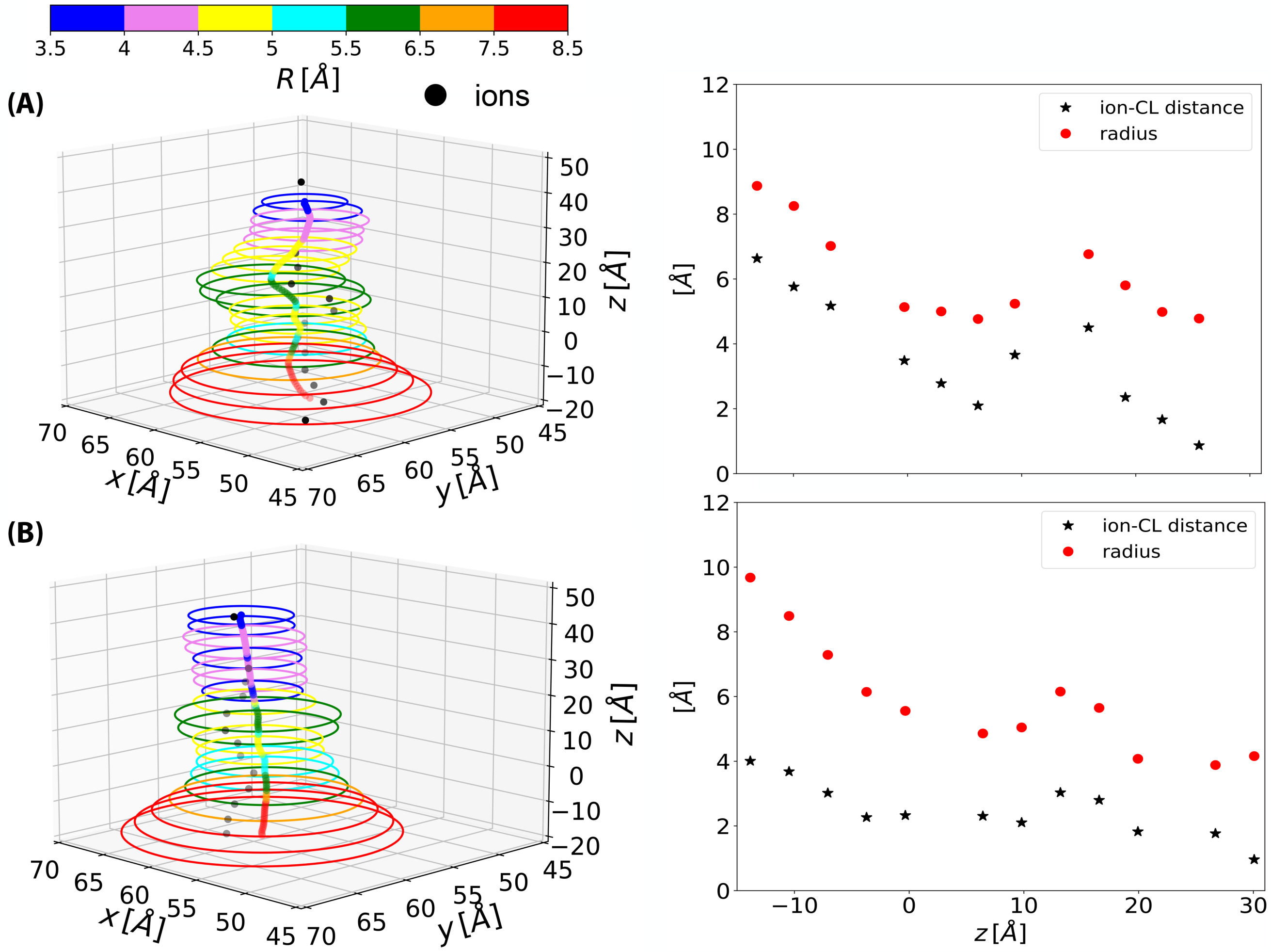

Figure S2. Averaged centerlines and ion trajectories, and their corresponding distances for the NL, **(A)**, and 1L systems **(B)**.

Fig. S2, illustrates in more detail how the trajectories of percolating Potassium ions, in the ion permeable systems NL and 1L, compare to the average MscL centerlines returned by Chanalyzer. The right-hand side of the figure shows the distances as a function of the z coordinate (within bins along z of about 3 $\AA$) between the ion positions and the centerline which are represented on the 3D plots on the left-hand side of the figure. These 3D plots are identical to those appearing in Fig. 4 **(A)** and **(B)** for NL (here top panel) and 1L (here bottom panel). The color code represents the effective radius returned by Chanalyzer whose relative circumferences projected on perpendicular planes to the z-coordinate are also shown. As discussed in the main text, steric effect cannot account alone for the deviations between the ion trajectory and the centerline–which is not uniform at different z positions even when the local radius is similar, without considering the presence of different net (negative) charges along the channel wall.
